# Self‐Assembly of Immune Signals to Program Innate Immunity through Rational Adjuvant Design

**DOI:** 10.1002/advs.202202393

**Published:** 2022-11-14

**Authors:** Michelle L. Bookstaver, Qin Zeng, Robert S. Oakes, Senta M. Kapnick, Vikas Saxena, Camilla Edwards, Nishedhya Venkataraman, Sheneil K. Black, Xiangbin Zeng, Eugene Froimchuk, Thomas Gebhardt, Jonathan S. Bromberg, Christopher M. Jewell

**Affiliations:** ^1^ Fischell Department of Bioengineering University of Maryland 8278 Paint Branch Drive College Park MD 20742 USA; ^2^ United States Department of Veterans Affairs VA Maryland Health Care System 10 North Greene Street Baltimore MD 21201 USA; ^3^ Department of Surgery University of Maryland School of Medicine Baltimore MD 21201 USA; ^4^ Center for Vascular and Inflammatory Diseases University of Maryland School of Medicine Baltimore MD 21201 USA; ^5^ Department of Microbiology and Immunology The University of Melbourne at the Peter Doherty Institute for Infection and Immunity Melbourne Victoria Australia; ^6^ Department of Microbiology and Immunology University of Maryland School of Medicine 685 West Baltimore Street Baltimore MD 21201 USA; ^7^ Robert E. Fischell Institute for Biomedical Devices 8278 Paint Branch Drive College Park MD 20742 USA; ^8^ Marlene and Stewart Greenebaum Cancer Center 22 South Greene Street Baltimore MD 21201 USA

**Keywords:** adjuvant, biomaterials, innate immunity, microparticles, nanoparticles, vaccine and immunotherapy

## Abstract

Recent clinical studies show activating multiple innate immune pathways drives robust responses in infection and cancer. Biomaterials offer useful features to deliver multiple cargos, but add translational complexity and intrinsic immune signatures that complicate rational design. Here a modular adjuvant platform is created using self‐assembly to build nanostructured capsules comprised entirely of antigens and multiple classes of toll‐like receptor agonists (TLRas). These assemblies sequester TLR to endolysosomes, allowing programmable control over the relative signaling levels transduced through these receptors. Strikingly, this combinatorial control of innate signaling can generate divergent antigen‐specific responses against a particular antigen. These assemblies drive reorganization of lymph node stroma to a pro‐immune microenvironment, expanding antigen‐specific T cells. Excitingly, assemblies built from antigen and multiple TLRas enhance T cell function and antitumor efficacy compared to ad‐mixed formulations or capsules with a single TLRa. Finally, capsules built from a clinically relevant human melanoma antigen and up to three TLRa classes enable simultaneous control of signal transduction across each pathway. This creates a facile adjuvant design platform to tailor signaling for vaccines and immunotherapies without using carrier components. The modular nature supports precision juxtaposition of antigen with agonists relevant for several innate receptor families, such as toll, STING, NOD, and RIG.

## Graphical abstract

Self‐assembly of peptide antigen and distinct combinations and ratios of toll‐like receptor agonists into immune polyelectrolyte multilayers reveal activating multiple toll‐like receptor signaling pathways generates distinct types of antigen‐specific response. This strategy can be used to improve vaccine design by initiating a multifaceted immune attack against a singular antigen creating robust antigen‐specific immunity.

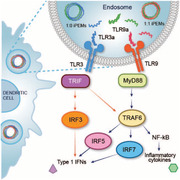

## 1 Introduction

The efficacy and durability of disease‐specific immune responses are determined not just by antigen specificity, but by features spanning T and B cell phenotype, antibody subclasses, cytokine secretion, tissue‐specific homing, and immune cell maintenance. Thus, vaccines and immunotherapies for nearly any disease would benefit from better control over the specific characteristics of immune responses they induce, not just from a larger magnitude of response.^[^ ^1^, ^2^ ^]^ For example, the vaccines for influenza and coronavirus are hindered by variable protection rates across the population, along with rapid mutations that lead to new variants.^[^ ^3^, ^4^ ^]^ In another context, cancer cells create microenvironments that directly suppress antitumor immune responses.^[^ ^5^ ^]^ Rationally designing vaccines or immunotherapies that elicit immune responses with specific characteristics could improve outcomes by generating responses that are potent and precise.^[^ ^6^ ^]^ However, even approved adjuvants do not exhibit well‐defined effects and the mechanism of action is poorly characterized.^[^ ^7^, ^8^ ^]^ These limitations hinder the understanding of how the composition of adjuvants connect to polarization of immunity.

Toward the goal above, a developing aspect of adjuvant research is combination adjuvant design and delivery. Toll‐like receptors (TLRs), for example, are proteins that have evolved to detect distinct pathogen associated molecular patterns (PAMPs) common in dozens of classes of pathogens. These PAMPs serve as TLR agonists (TLRas), initiating distinct innate and adaptive signaling cascades to drive immunity.^[^ ^9^ ^]^ Individually, TLRas are being exploited as adjuvants in numerous preclinical and clinical approaches.^[^ ^10^ ^]^ However, an important new discovery is that combinations of TLRas can generate adjuvant functions that are distinct and synergistic from the individual TLRas.^[^ ^11^, ^12^ ^]^ For example, a candidate vaccine composed of melanoma peptides, TLR4a, and TLR3a increases T cell responses in late stage melanoma patients.^[^ ^13^ ^]^ In an important preclinical example, delivery of polymer particles co‐loaded with TLR4a and TLR7a provided protective immunity against lethal avian and swine flu virus challenges.^[^ ^14^ ^]^ This TLRa combination activated distinct pathways, and protective immunity required co‐loading of signals in the same particle. More recently, this theme of biomaterial‐enabled delivery of multiple TLRas has shown promise in a number of immune applications.^[^ ^13^, ^15^, ^16^ ^]^ This body of work illustrates the potential of co‐delivery of TLRas to activate multiple lymphocyte compartments (i.e., T and B cells) to enhance immunity.

Biomaterials are being explored to improve adjuvants because these materials offer attractive features, such as co‐delivery of multiple cargos and formation of easily internalized particles.^[^ ^17^ ^]^ While biomaterials have traditionally been viewed as passive carriers, many studies now reveal that some of the most ubiquitous polymeric carriers can activate TLRs, inflammasomes, and other stimulatory immune pathways, even in the absence of antigens, adjuvants, or other signals.^[^ ^18^, ^19^ ^]^ Thus, while polymeric materials offer great potential, these intrinsic immune characteristics can complicate vaccine design because the carrier itself may alter how immune signals are received or processed. In addition, the complexity of material systems often hinder regulatory assessment and approval during the chemistry and manufacturing controls (CMC) phase.^[^ ^20^ ^]^ Finally, from an immunological perspective, potent and efficient responses are driven by high density display of immune signals. Thus, embedding or encapsulating immune cues in polymer or lipid matrix effectively dilutes the mass loading percentage of these signals.

To mimic the attractive features of biomaterials—such as co‐delivery, but avoid the limitations of traditional nanomaterials outlined above, we developed immune polyelectrolyte multilayers (iPEM). iPEMs are self‐assembled capsules built entirely from immune signals through electrostatic interactions. Intriguingly, the modular aspect of iPEMs provides a unique opportunity for rational design of combination adjuvants that program specific features for elicitation of the immune responses. Furthermore, because 100% of the “carrier” is cargo, iPEMs juxtapose immune signals at high density. We previously showed a peptide antigen and a single TLRa remain accessible to immune cells following assembly into iPEMs.^[^ ^21^, ^22^ ^]^ Here we exploited the modularity of this platform to create libraries of multiple classes of TLRas and peptide antigens. We show iPEMs recruit and enrich TLRs in primary immune cells as a function of the cognate agonists in iPEMs. The composition of iPEMs enables control over the type and relative level of simultaneous TLR signaling and gene expression across multiple TLR pathways during antigen presentation. This control allows generation of different types of T cell response to the same antigen and promotes pro‐immune stromal microenvironments in secondary lymphoid organs of mice vaccinated with iPEMs. These changes ultimately result in enhanced T cell function and anti‐tumor immunity during vaccination with iPEMs containing multiple classes of TLRa, but not with other dose‐matched iPEM designs or ad‐mixed formulations. There is great need in vaccine and immunotherapy applications for flexible platforms that allow rapid integration of combinations of antigens or immunotherapies without redesign. Thus, the precision control iPEMs provide over innate immune signaling could contribute to more specific, rapid, and efficient design of vaccines and immunotherapies.

## 2 Results and Discussion

### 2.1 iPEMs Allow Assembly of Multiple TLR Agonist Classes at Defined Ratios

**Figure 1:**
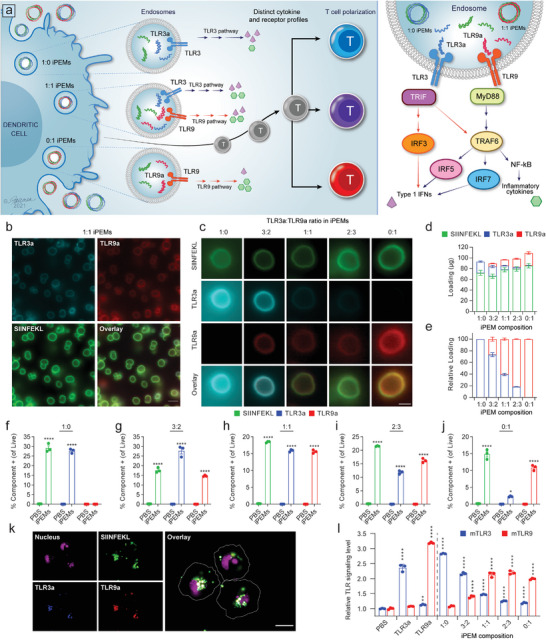
a) iPEMs synthesized with distinct ratios of TLRas differentially polarize the immune response. b,c) Representative fluorescence microscopy images of iPEMs built from different ratios of TLR3a and 9a ranging from 0% to 100% at a fixed total concentration. Scale bar is 5 µm in (b) and 2 µm in (c). d) The tunable absolute yield and e) relative composition of TLRas in a batch of iPEMs (*n* = 12 biological replicates). f–j) Percentage of cells positive for iPEM signals due to uptake after treatment with iPEMs with different ratios of TLRas (*n* = 3 biological replicates, stats shown are comparison to PBS control). k) Representative fluorescence microscopy images of iPEM uptake by antigen‐presenting cells. Scale bar is 30 µm. l) TLR reporter cells activity at 16 h as a function of TLRa ratio in iPEMs. Error bars are SEM. Statistical analysis was done by a two‐way ANOVA with a Tukey post test to correct for multiple comparisons. n.s.: not significant, *p < 0.05, **p < 0.01, ***p < 0.001, ****p < 0.0001.

We sought to determine if juxtaposition of antigen and combinations of TLRas in iPEMs drives efficient responses, while the relative composition of TLRas programs the type of response (**Figure** **1**a). We first tested if a library of iPEMs could be assembled from a common model peptide antigen derived from ovalbumin (SIINFEKL) and defined combinations of TLRas spanning different TLR pathways and structural features. These pathways included a double stranded RNA agonist for TLR3 (polyIC) and a single stranded DNA agonist for TLR9 (CpG). By fixing the total amount of antigen and total TLRa – but using defined relative input ratios of TLR3a:TLR9a, iPEMs could be readily assembled to contain each component within the same bilayers (Figure 1b). Importantly, the TLRa composition could be controlled over the entire range from pure TLR3a (i.e., 1:0 TLR3a:TLR9a) to pure TLR9a (i.e., 0:1 TLR3a:TLR9a) (Figure 1c). Likewise, quantitative loading measurements demonstrated maintenance of stable antigen concentration (Figure 1d), while directly controlling the relative concentrations of TLR3a and TLR9a (Figure 1e). This data confirmed iPEMs can be used to assemble antigen and multiple immune signals without carrier components. Most pathogens harbor multiple PAMPs, and in natural infections, immunity is strongly amplified by several PAMPs presented simultaneously on particulate moieties (e.g., viruses or bacteria).^[^ ^23^ ^]^ To mimic this combination, adjuvants with multiple PAMPs have shown promise in recent vaccine research. For example, recent studies have shown adjuvants simultaneously triggering multiple pathogen recognition receptors—such as TLRs, to enhance cytokine secretion and drive more robust cellular and humoral responses in tumor models and clinical trials.^[^ ^10^ ^]^ While the focus of this paper is on distinct combinations of TLRas, the modularity of the iPEM platform would also allow for other types of PAMPs to be incorporated, as long as these cues are charged or can be modified to be charged. Some signaling families that would be of particular interest for vaccine adjuvants in this respect include NOD‐like agonists, RIG‐like agonists, or STING agonists. Additional chemistries might also allow for incorporation of other molecular classes, such as small molecule adjuvants (e.g., MPLA, imidazoquinolines).

### 2.2 iPEMs Co‐Deliver Multiple Immune Cues to Primary DCs for Uptake

Dendritic cells (DCs) in secondary lymphoid organs, such as spleen and lymph nodes (LNs), integrate combinations of signals to prime lymphocytes and then direct the differentiation and fate of these cells to determine the phenotype of the immune response. We tested if iPEM composition could control the levels of each signal co‐delivered to splenic DCs (CD11c^+^). Following iPEM treatment, flow cytometry revealed DCs exhibited clear fluorescent signal corresponding to each iPEM composition from fluorescently tagged components, and excitingly, iPEMs containing a combination of TLR3a and TLR9a caused increasing or decreasing signal for each component, depending on the relative ratio of TLR3a:TLR9a in iPEMs (Figure 1f–j). Fluorescence microscopy revealed iPEMs were taken up by DCs into the intracellular region resulting in spatial co‐localization of peptide antigen (green) and TLRas (blue or red) (Figure 1k). Presenting TLRas in particulate form for intracellular delivery is an important capability that could improve the pharmacokinetics in vivo because one of the major challenges in translating the outcomes of TLRas based immunotherapeutics has been the use of soluble agonists. For example, small molecule adjuvants and hydrophilic adjuvants are easily diffusible and can quickly move from the site of administration into the systemic circulation, leading to cytokine storm and severe adverse inflammatory responses.^[^ ^24^ ^]^ These limitations can be even more challenging with multiple TLRas. We have previously shown that iPEMs are taken up more efficiently than soluble TLRas.^[^ ^22^ ^]^ Using nanotechnology for structural optimization and display of cues at high density and with specific ratios could enable localized triggering of families of TLRs for new adjuvants.

### 2.3 iPEM Composition Dictates the Relative Levels of TLR Signaling

Since the ratio of TLRas in iPEMs correlated with signal uptake, we tested if TLR signaling would be correspondingly controlled by this design parameter using a quantitative TLR reporter system. As hypothesized, iPEMs containing pure TLR3a (1:0) only activated TLR3, while iPEMs containing only TLR9a (0:1) activated only TLR9 reporters (Figure 1l). Excitingly, iPEMs containing both TLR3a and TLR9a activated pathways at levels that were dependent on the relative loading level of each agonist in the iPEMs. Together, the data in Figure 1 reveals iPEMs allow programmable control over the relative uptake of multiple adjuvant cargo classes, and the corresponding levels of TLR activation for each of the TLR ligands included in the iPEM design.

### 2.4 iPEMs Sequester Cognate TLRs and Initiate Antigen Presentation Following Internalization through Endosomal/Lysosomal Pathways

**Figure 2:**
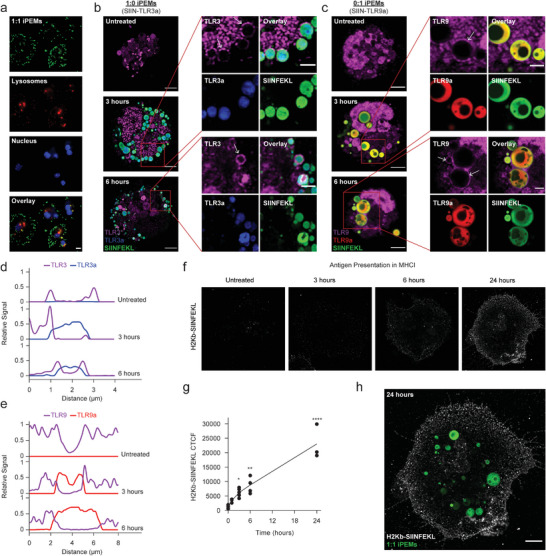
iPEMs deliver TLRa cargo to their spatially restricted cognate receptors. a) Representative fluorescence microscopy images of iPEM uptake by antigen‐presenting cells showing colocalization with lysosomes at 3 h. Scale bar is 10 µm. b,c) Representative images of iPEMs interfacing with their cognate receptors intracellularly. Scale bar is 5 µm for main image and 2 µm for inset. Line trace profiles showing the relative signal intensity of d) TLR3 versus TLR3a and e) TLR9 versus TLR9a. f) Representative images of antigen presentation over time following iPEM treatment. g) CTCF of antigen presentation over time. h) Representative images of antigen‐presentation on the surface of primary DCs following iPEM treatment. Scale bar is 5 µm. Error bars are SEM. Statistical analysis was done by a two‐way ANOVA with a Tukey post test to correct for multiple comparisons. n.s.: not significant, *p < 0.05, **p < 0.01, ***p < 0.001, ****p < 0.0001.

We next tested if iPEMs concentrate TLRa in endolysosomes, where many spatially‐restricted TLRs are displayed.^[^ ^25^ ^]^ Incubation of iPEM‐treated DCs with lysosomal stains revealed association of iPEMs with the cell membrane, and within 3 hours, iPEMs beginning to concentrate in lysosomes (**Figure** **2**a). Image quantification revealed over 80% of area positive for iPEM signal was also positive for lysosome signal, indicating colocalization (Figure S1a, Supporting Information). This occurred regardless of composition, confirming iPEMs concentrate TLRa cargo used to build iPEMs in locations where the target receptor is expressed. Interestingly, approximately 20% of iPEMs were not co‐localized with lysosome, indicating at least some fraction of iPEMs reached the cytosol; we observed some of these iPEMs in the intracellular space, and also localized near the nucleus. This discovery suggests application of iPEMs to receptor families requiring cytosolic engagement—such as STING or RIG‐1—could be an interesting area for future studies on combinatorial adjuvant delivery. We next examined if iPEMs colocalizing TLRa in endolysosomes promoted co‐localization with distinct TLRs. Using intracellular stains for each TLR (i.e., the receptors) over time and iPEMs containing a single TLRa, we discovered 1:0 or 0:1 iPEMs taken up by DCs interfaced with TLRs (Figure 2b,c). Intriguingly, over 6 h we observed recruitment and enrichment of the corresponding receptors—TLR3 and TLR9—around regions rich in the iPEMs containing either TLR3a or TLR9a, respectively (Figure 2b,c, arrowheads). This was further confirmed by quantitative image analysis and signal intensity profiles along the endosome structures (Figure 2d,e; Figure S1b, Supporting Information). The expression and trafficking of TLRs is known to be regulated by a variety of factors. While at rest, TLRs are typically found to be endoplasmic reticulum (ER)‐associated;^[^ ^26^ ^]^ the data above indicate recruitment or enrichment of TLRs on the surface of endosomes. Activation of TLRs is one of the ways in which TLRs can be trafficked to endosomes from the ER. The recruitment of TLRs to endolysosomes containing iPEMs or other TLRa‐containing moieties could thus drive activation of TLR signaling and consequently more robust immune responses.

We next tested if the kinetics of iPEM‐driven TLR engagement aligned with presentation of SIINFEKL antigen in MHC class I antigen presentation machinery (MHCI). Both confocal microscopy and image analysis revealed increasing antigen presentation on the cell surface over time (Figure 2f–h), with notable increases over the first 6 h, the interval over which TLR recruitment was observed (Figure 2b,c). We further confirmed this by image analysis quantifying the Corrected Total Cell Fluorescence (CTCF) of antibody that binds to SIINFEKL antigen presented in MHCI. This analysis revealed a significant increase in antigen presentation over time (Figure 2g). Importantly, the presence of internalized antigen—indicated by FITC labeled SIINFEKL, at the same time antigen was displayed on the surface—indicated by H‐2K^b^ SIINFEKL, confirmed antigen was processed from iPEMs and efficiently presented (Figure 2h). This further indicated that iPEM components were capable of crossing the lipid‐bilayers which would allow for delivery of RNA or cytosol‐active immunostimulants using the iPEM platform. Some exciting possibilities include mRNA encoding vaccine components, or other classes of noncoding nucleic acids, such as agonists for pattern recognition receptors (e.g., RIG‐I and STING pathways). Taken together, these data suggest following internalization, iPEMs concentrate and sequester TLRs binding their cognate ligands, initiating antigen processing and presentation.

### 2.5 iPEMs Selectively Activate TLR Signaling Pathways to Alter Gene Expression Depending on iPEM Composition

**Figure 3:**
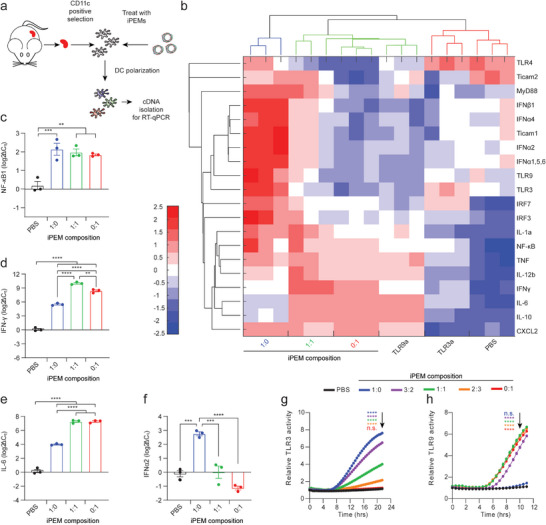
iPEMs activate TLR signaling pathways as a function of composition. a) cDNA was isolated from primary DCs cultured with iPEMs. b) Unsupervised hierarchal clustering of RT‐qPCR analysis of gene expression. Gene expression in the heat map is standardized for comparison across multiple genes. Each biological replicate (a single column of the heatmap, *n* = 3 per treatment) is an aggregation of *n* = 7 technical treatment replicates. c) NF‐*κ*B, d) IFN*γ*, e) IL‐6, and f) IFN*α*2 expression changes relative to PBS control. g) TLR3 reporter cell activity over time, h) TLR9 reporter cell activity over time (*n* = 3 biological reps for each). Error bars are SEM. Statistical analysis was done by a two‐way ANOVA with a Tukey post test to correct for multiple comparisons. n.s.: not significant, *p < 0.05, **p < 0.01, ***p < 0.001, ****p < 0.0001. Statistics shown for time point indicated by arrows.

We next quantified key genes in the TLR signaling pathway by RT‐qPCR to test if iPEMs allow combinatorial control of TLR‐mediated gene expression, depending on the combination and ratio of TLRa assembled with antigen (**Figure** **3**a). Unsupervised hierarchal clustering of expression in iPEM‐treated DCs revealed clear segmentation between iPEM designs and controls (Figure 3b). Clustering further organized iPEM treatments in order of decreasing TLR3a:TLR9a ratio, indicating a functional correlation based on the composition of TLRas in iPEMs. All iPEMs significantly increased NF‐*κ*B (Figure 3c), a transcription factor critical in expression of pro‐inflammatory cytokines that shape immunity.^[^ ^27^ ^]^ Although TLR3 and TLR9 engagement involve different signaling pathways, both lead to NF‐*κ*B.^[^ ^28^ ^]^ We also assessed gene expression of several cytokines indicative of specific TLR signaling cascades: IFN*γ* (Figure 3d) induced by both TLR3a and TLR9a following NF‐*κ*B production (Figure 3d), IL‐6 induced primarily by TLR9a (Figure 3e), and IFN*α*2 induced primarily by TLR3a (Figure 3f).^[^ ^29^ ^]^ iPEMs containing high ratios of TLR3a:TLR9a significantly increased IFN*α*2 compared to control and to lower ratios of TLR3a:TLR9a (Figure 3e), consistent with type I IFN induction by the TLR3 signaling pathway.^[^ ^30^ ^]^ In these studies, iPEMs with a low ratio of TLR3a:TLR9a drove higher levels of IL‐10 and IL‐6 gene transcription compared to the negative control and relative to the higher TLR3a:TLR9a ratio (Figure 3e; Figure S2a, Supporting Information). As the TLR3a:TLR9a ratio increased, the gene expression pattern changed, with TNF*α* and IL‐1*α* gene expression increasing to higher levels for iPEMs with more TLR3a (Figure S2b,c, Supporting Information). Interestingly, the highest levels of IFN*γ* were induced by iPEMs with a combination of both TLR3a and TLR9a compared to a single TLRa (Figure 3d). This indicates synergy associated with activating multiple TLRa pathways. Of note, iPEMs also induced higher gene expression levels compared to the soluble controls. Future studies will investigate the role of biophysical presentation form on TLR signaling. Since iPEMs significantly increased expression of genes involved in TLR signaling, we next investigated the effect of TLR ratio on kinetics of signaling using a TLR reporter system. In these studies, the level of activation was dependent on the amount of each TLRa in the iPEMs (Figure 3g,h). With respect to kinetics, iPEMs containing higher ratios of TLR3a:TLR9a increased TLR3 activity faster than iPEMs with lower ratios of TLR3a:TLR9a (Figure 3g), while iPEMs with lower ratios of TLR3a:TLR9a activated TLR9 more quickly (Figure 3h). This demonstrated iPEMs allow control over both the levels and kinetics of TLR activation—and the associated downstream gene expression – by defining the ratios of TLRa assembled with antigen. The particular ratios we selected for these studies were from a large library we created comprised of higher and lower ratios. We chose this range to assess the entire spectrum and demonstrate a range of outcomes. Although distinct antigen presenting cell (APC) subsets are known to have differential expression of TLRs, the TLRas selected for this study are highly expressed across many mouse and human APC subsets, so these results are representative of both mouse and human APC activation. From a practical perspective, this approach supports a system for rapidly integrating an antigen of interest with flexibility to generate divergent immune outcomes by controlling innate signaling.

Activation of TLRs on DCs triggers antimicrobial activity together with distinct inflammatory patterns depending on the TLR agonist encountered; these processes modulate the subsequent adaptive immune response. In our study, the engagement of TLR9 with TLR9a in iPEMs upregulated the production of mRNA encoding cytokines IL‐6 and IL‐10, while engagement of TLR3 primarily increased the generation of type I interferons associated with antiviral response. These findings support the connection between iPEM composition and the type of resulting immune function. For example, polyIC is a double‐stranded RNA that mimics and warns against viral pathogens. Accordingly, we found IFN*α*2, which is important in initiating antiviral responses, increased with the ratio of polyIC:CpG (TLR3a:TLR9a) in iPEMs. However, many cytokines have multifaceted roles in other disease settings that could also create immunotherapeutic opportunities.^[^ ^31^, ^32^, ^33^, ^34^, ^35^ ^]^ For example, although IL‐10 has anti‐inflammatory features that can favor tumor escape, IL‐10 has also been reported to mediate regression of established melanoma and breast cancer metastases in preclinical models.^[^ ^31^ ^]^ As another illustration, IL‐6 can promote Th2 differentiation and simultaneously inhibit Th1 polarization through independent mechanisms.^[^ ^32^ ^]^ The success of the monoclonal antibody tocilizumab—which targets the receptor for IL‐6 (IL‐6R)—in the treatment of inflammatory arthritis has identified IL‐6 as a key cytokine in these processes.^[^ ^33^ ^]^ While here we focus on TLR pathways, a number of other intersecting innate pathways—such as NOD, RIG‐I, and STING, could also be exploited in a modular way. An important question for such future studies would be consideration of shared transduction pathways across these innate pathway families.^[^ ^36^ ^]^ Thus, the ability to induce multiple cytokines with distinct immune function and to program their relative levels in vitro or in vivo could be exploited as a tool to elucidate the distinct roles of combinatorial adjuvant components, or to support new drug candidates.

### 2.6 Assembling iPEMs from Multiple TLRas Drives Synergistic DC Activation

**Figure 4:**
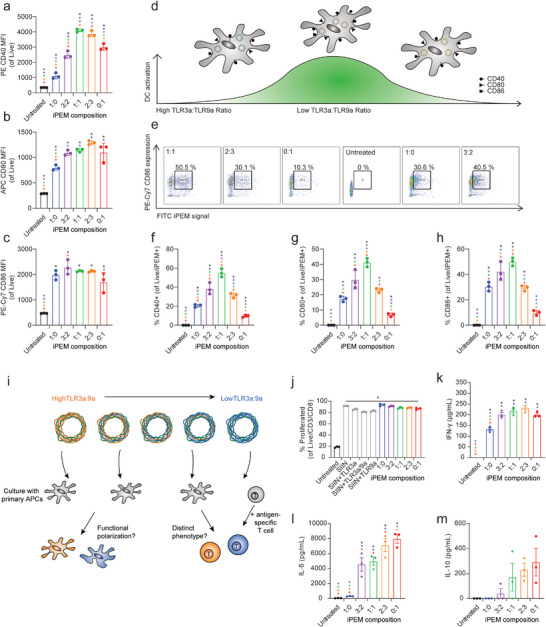
iPEMs activate DCs as a function of composition. a–c) Flow cytometry analysis of the mean fluorescence intensity of surface markers of DC activation (*n* = 3 biological replicates). d) Schematic of DC activation following iPEM treatment. e) Representative flow plot. f–h) Flow cytometry analysis of DC activation as a function of iPEM uptake (*n* = 3 biological replicates). i) iPEMs generate different immune responses against the same antigen. j) Percent proliferation of antigen‐specific T cells. k) IFN*γ* secretion by antigen specific T cells. l) IL‐6 secretion by antigen specific T cells. m) IL‐10 secretion by antigen‐specific T cells. Statistical analysis was done by a two‐way ANOVA with a Tukey post test to correct for multiple comparisons. Error bars are SEM. *p < 0.05. Color of * indicates comparison to that respective color's group.

We next tested if the ability of iPEMs to co‐deliver multiple classes of signals enhances DC activation relative to a single TLRa. We again fixed total antigen and TLRa, but varied the relative TLRa ratio. We then assessed activation using expression of common costimulatory molecules, including CD40, CD80, and CD86. All iPEM designs significantly increased activation markers on live cells compared to untreated cells (**Figure** **4**a–c; Figure S3a, Supporting Information). Interestingly, for two activation markers, statistically significant synergies were associated with iPEMs containing two classes of TLRas. For example, for CD40 and CD80, the 1:1 and 2:3 ratios, respectively, drove the highest expression of all designs. Thus, we hypothesized the level of activation correlates with the number and combination of iPEMs internalized at the single cell level (Figure 4d). To test this, we measured the percentage of cells positive for each activation marker among cells positive for each iPEM composition using the FITC‐SIINFEKL as an indicator for iPEMs (Figure 4e; Figure S3b,c, Supporting Information). Excitingly, this data revealed activation for all markers is the highest in cells internalizing iPEMs containing multiple classes of TLRa (e.g., 1:1 TLR3a:TLR9a) (Figure 4f–h). Thus, incorporating two immunostimulants into the same iPEM allows for the juxtaposition of signaling within a given cell to provide direct control over the relative levels of TLR signaling in the cell internalizing the iPEM. This is significant because although a simple mixture of monovalent iPEMs could allow for activation of multiple pathways, this design lacks the ability to control the ratio of signals a specific cell receives; this integration is a key parameter that determines the nature of immune response and differentiation.

### 2.7 iPEM Composition Allows Induction of Different T cell Responses to the Same Antigen

Based on the tunable control of DC function shown above, we hypothesized that different types of T cell responses could be generated against the same antigen in a programmable way by selecting the combination of innate cues (i.e., TLR ligands) included in iPEMs that DCs encounter. We treated DCs with iPEMs and co‐cultured these cells with transgenic T cells (OT‐I) specific for SIINFEKL presented in MHC class I (Figure 4i). These T cells proliferate and differentiate depending on the other cues encountered (e.g., co‐stimulatory signals, cytokines). In a common fluorescence dilution assay, we observed significantly increased proliferation in all samples containing SIINFEKL antigen, either soluble or incorporated into iPEMs (Figure 4j; Figure S3d, Supporting Information). This was indicated by greatly reduced MFIs resulting from successive dilution during T cell division (Figure S3e, Supporting Information). Assessing the function of these T cells for cytokine secretion by ELISA revealed that treatment with iPEMs containing high ratios of TLR3a:TLR9a significantly increased IFN*γ* (Figure 4k), without increasing IL‐6 (Figure 4l) or IL‐10 (Figure 4m), confirming the gene expression results in Figure 3. Strikingly, as the TLR3a:TLR9a ratio decreased—while IFN*γ* remained elevated, IL‐6 levels significantly increased in a manner that correlated with the TLRa ratio. For IL‐10, the differences did not reach statistical significance when comparing the various iPEM formulations, but the trend was similar: decreasing ratios of TLR3a:TLR9a were associated with an increase in IL‐10. Notably, these outcomes are aligned with the nature of TLR3a (viral) and TLR9a (bacterial) since IL‐6 is important in promoting antibody production—useful to combat bacterial infections—by B cells, while several IFN*γ* functions are important in combating viral infection (e.g., increased antigen presentation).^[^ ^37^ ^]^ Furthermore, the highest levels of IFN*γ* were measured from cells treated with iPEMs containing a combination of two TLRas, which was significant compared to iPEMs containing TLR3a alone (Figure 4k). Taken together, these results indicate iPEMs can generate distinct antigen‐specific responses against the same antigen by altering the compositions of TLRas. This creates potential for rational control of immune signaling to enable specific adjuvant profiles.

### 2.8 iPEM Vaccination Alters the Composition, Activation State, and Structure of Draining LNs

**Figure 5:**
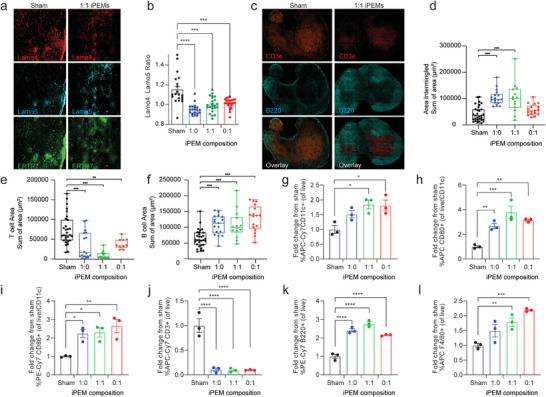
iPEM vaccination in mice changes the activation state, composition, and structure of the draining LN. a) Representative fluorescence microscopy images of LN sections immunohistochemically stained with ERTR7, laminin‐*α*4, and laminin‐*α*5. b) Ratio of laminin‐*α*4 to laminin‐*α*5 calculated from image analysis of stained sections. c) Representative fluorescence microcopy images of LN sections immunohistochemically stained with CD3 and B220. d) Area of T cells and B cells intermingled calculated from image analysis of stained sections. e) Area of T cells calculated from image analysis. f) Area of B cells calculated from image analysis. g) Frequency of CD11c^+^ cells relative to untreated. h) Frequency of CD80^+^ cells relative to untreated. i) Frequency of CD86^+^ cells relative to untreated. j) Frequency of CD3^+^ cells relative to untreated. k) Frequency of B220^+^ cells relative to untreated. l) Frequency of F4/80^+^ cells relative to untreated. *n* = 3 biological replicates. Statistical analysis was performed by a two‐way ANOVA with a Tukey post test to correct for multiple comparisons. Error bars are SEM. n.s.: not significant, *p < 0.05, **p < 0.01, ***p < 0.001, ****p < 0.0001.

We next examined the role activation of multiple TLR signaling pathways has on the structure and function of LNs following vaccination. The LN microenvironment is complex, containing immune cells, along with stromal cell and tissue components that work together to coordinate immunity.^[^ ^18^ ^]^ During an immune response, T cell and B cell zones are reorganized to promote interactions between APCs, T cells, and B cells. This is facilitated by changes in the stromal structure of the LN, such as changes in the expression of extracellular matrix proteins that can control cellular interactions. We vaccinated C57BL6/J mice subcutaneously at the tail base with either PBS (Sham) or iPEMs containing specific combinations of TLRas and analyzed the draining LN (dLN). Using immunohistochemistry, we stained sections for ERTR7—a fibroblastic reticular cell marker—to visualize the overall morphology and structure of the LN. We also stained laminin‐*α*4 and laminin‐*α*5, important extracellular matrix proteins have been implicated in strong immunity—when the ratio of laminin‐*α*4:laminin‐*α*5 decreases, and tolerance—when the ratio of laminin‐*α*4:laminin‐*α*5 increases (**Figure** **5**a).^[^ ^38^ ^]^ These studies revealed a significant reduction in the ratio of laminin‐*α*4 to laminin‐*α*5 in iPEM‐treated cohorts relative to sham (Figure 5b). This shift in stromal structure indicates all iPEM designs polarize the local LN environment toward a pro‐immune state. We next analyzed the dLN for intermingling of T cell and B cell zones (Figure 5c). This analysis indicates a significant increase in lymphocyte intermingling following iPEM vaccination, further supporting an activated LN state (Figure 5d). Likewise, analysis of T and B cell area revealed a decrease in T cell areas and an increase in B cell areas, reflecting increased intermingling as a result of iPEM vaccination (Figure 5e,f).

To determine how iPEMs alter the activation state of dLNs at the cellular level, we vaccinated mice on days 0 and 14. Analysis of conventional CD11c+ DCs revealed iPEMs increased the frequency of DCs (Figure 5g), as well as their activation, indicated by increased expression of CD80 (Figure 5h) and CD86 (Figure 5i). This confirmed that iPEM vaccination stimulated the activation state of DCs in the dLN. We next assessed the change in B cell and T cell areas indicated by histology, and used flow cytometry to quantify the frequency of T cells (CD3) (Figure 5j), B cells (B220) (Figure 5k), and macrophages (F4/80) (Figure 5l) in dLNs. Confirming the histologic studies (Figure 5e,f), iPEMs caused a decrease in T cell frequency (Figure 5j) while B cells increased (Figure 5k). We additionally found an increase in the frequency of macrophages in LNs of mice vaccinated with iPEMs (Figure 5l). Overall, these data revealed iPEMs promote a pro‐immune LN environment, increasing APC frequencies and activation state, and enhancing the intermingling of T and B cells.

### 2.9 iPEM Vaccination Increases the Frequency and Preferential Trafficking of Antigen Specific T Cells

**Figure 6:**
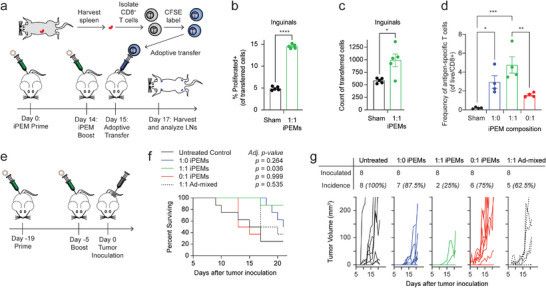
Vaccination with iPEMs containing multiple TLRas expand antigen‐specific T cells and confer synergistic efficacy. a) Schematic of adoptive transfer. b) Proliferation of antigen‐specific T cells (Day 17) after prime and boost iPEM vaccinations on Day 0 and Day 14, respectively, in the draining (inguinal) LN. c) Count of CFSE+ T cells in the draining (inguinal) LN following adoptive transfer (Day 17). d) MHC‐I SIINFEKL tetramer staining of antigen‐specific T cells in blood on Day 21. *n* = 5 biological replicates for (a–c) and *n* = 4 biological replicates for (d). Statistical analysis was assessed using Student's two‐tailed *t*‐test for (b, c) and by a two‐way ANOVA with a Tukey post test to correct for multiple comparisons for (d). Error bars are SEM. n.s.: not significant, *p < 0.05, **p <0.01, ***p < 0.001, ****p < 0.0001. e) Schematic of iPEM vaccination regimen (days −19, −5) and tumor inoculation (day 0). f) Kaplan–Meier survival curve at Day 21 with exact *p*‐values from a log‐rank test compared to the untreated control and adjusted for multiple comparison (Sidàk). g) Line traces for all mice in the study with the number of observed tumors (visual or palpable) indicated above the graph for each cohort. Cohort sizes were *n* = 8 and no mice were excluded.

To determine if the local changes induced by iPEMs result in recruitment and expansion of antigen‐specific T cells, we vaccinated mice and adoptively transferred CFSE‐labeled OT‐I T cells (**Figure** **6**a; Figure S4a, Supporting Information). These cells exhibit T cell receptors specific for SIINFEKL, thus there is a large pool of naïve T cells to study. This is a common tool that enables facile tracking of proliferation and migration of antigen‐specific T cells. We chose to test the 1:1 iPEMs as the results from the previous studies revealed the largest differences in this treatment group. Two days after transfer, the dLN (inguinal) and non‐dLN (axillary, Figure S4b,c, Supporting Information) were harvested and analyzed for the presence and proliferation of the transferred antigen‐specific T cells. We discovered a significant increase in proliferation of these T cells in the dLN and non‐dLN of iPEM‐vaccinated mice (Figure 6b; Figure S4b, Supporting Information), as well as increased numbers of transferred T cells in the dLN, but not in the non‐dLN (Figure 6c; Figure S4c, Supporting Information). These findings indicate iPEMs recruit antigen‐specific T cells and stimulate expansion of these antigen‐specific cells migrating to the dLN after adoptive transfer. In these sites the migrating T cells engage APCs activated by iPEMs to mount antigen‐specific responses. During vaccination, vaccines must activate and expand antigen‐specific T cells from within the total T cell repertoire of the host. Thus, we next returned to the full set of iPEMs ratios used earlier, 1:1, 1:0, and 0:1 during a classic prime‐boost vaccination regimen. In this study, mice were primed with iPEM vaccination on Day 0 and boosted on Day 14. On Day 21, the frequency of antigen‐specific T cells was analyzed using MHC‐I tetramers. One week post boost, we observed significant expansion of antigen‐specific T cells for all iPEM designs relative to sham (Figure 6d). Excitingly, 1:1 iPEMs generated the highest level of statistical difference compared with 1:0 or 0:1 iPEMs, indicating synergy associated with activating multiple TLR pathways.

### 2.10 iPEMs Containing Multiple TLRa Provide Synergistic Efficacy

We next sought to assess how the enhanced T cell function driven by iPEMs containing multiple classes of TLRas impacts anti‐tumor immunity in a preclinical cancer model. In these studies, we hypothesized iPEMs containing both TLRas would provide robust innate signaling that maximized efficacy. Thus, mice received priming and booster vaccinations using formulations containing fixed doses of antigen and total TLRa, but different relative amounts of TLRa (Figure 6e). Prime boost regimens are used to capitalize on the memory and exquisite specificity of the adaptive immune system; these features generally create larger recall responses after a booster injection, relative to the primary response after an initial injection. In these studies, cohorts received i) 1:0 iPEMs, ii) 1:1 iPEMs, iii) 0:1 iPEMs, or iv) an ad‐mixed formulation of CpG, polyIC, SIINFEKL (without anchor). The last cohort was included to test the hypothesis that nanostructured juxtaposition of signals is important for efficacy, while the first three cohorts test the hypothesis that a combinatorial adjuvant formulation improves immunogenicity. Mice were inoculated with B16 tumor cells expressing OVA 5 d after the booster vaccination and monitored for tumor growth and survival. Excitingly, 1:1 iPEMs conferred strong efficacy, indicated by both tumor progression (Figure 6f) and incidence (Figure 6g). In contrast, neither of the iPEMs containing a single TLRa provided statistically significant improvements, nor did the ad‐mixed formulations. Together, this data reveals that iPEMs generate superior antitumor immunity as a result of both i) inclusion of multiple classes of TLRas and ii) high density juxtaposition of these cues with antigen in a defined manner.

### 2.11 iPEMs Allow Assembly of Human Relevant Melanoma Antigen with Combinatorial Libraries of TLR Agonists

**Figure 7:**
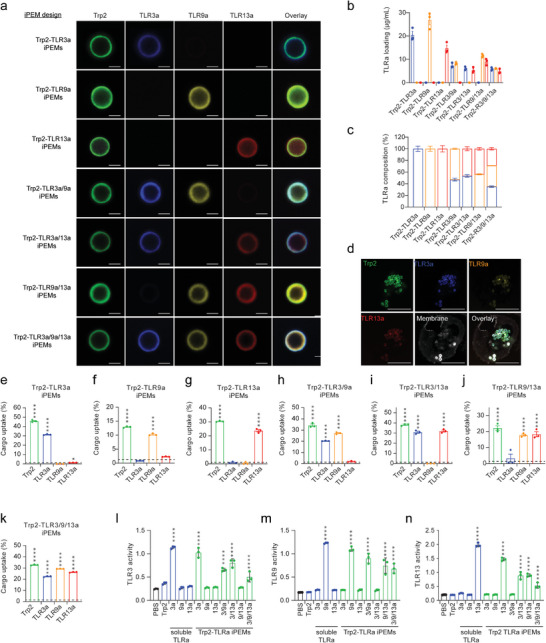
iPEMs are modularly self‐assembled from one or multiple TLRas with human tumor antigen. a) Representative fluorescence microscopy images of iPEMs built entirely from three bilayers of Trp2 and only one class of TLRas or combination of any two or three TLRas (green, Trp2; blue, TLR3a; red, TLR9a; yellow, TLR13a; scale, 5 µm). b) Absolute loading of TLRas in each type of iPEM. c) Relative loading of TLRas in each composition of iPEMs. d) Intracellular colocalization of Trp2 and TLR3a, TLR9a and TLR13a visualized by confocal microscopy. Quantification of intracellular uptake of iPEMs assembled from Trp2 and either one class of e–g) TLR3a, 9a, or 13a, or h–j) iPEMs from combination of any two of the three TLRas, or k) iPEMs from triple combination of these three classes of TLRas. Activation of l) TLR3, m) TLR9, and n) TLR13 signaling in separate cultures of HEK‐Blue TLR3, TLR9, and TLR13 reporter cells following a 16 h incubation with iPEMs or controls. *n* = 3 biological replicates for all studies. Statistical analysis was done by a two‐way ANOVA with a Tukey post test to correct for multiple comparisons. Error bars are SEM. n.s.: not significant, *p < 0.05, **p < 0.01, ***p < 0.001, ****p < 0.0001.

We hypothesized the modular nature of iPEMs would enable assembly of more diverse combinations of immune cues, including three different classes of TLRas and a conserved human melanoma antigen (Trp2). Using an analogous assembly process, we were able to assemble iPEMs with high fidelity using Trp2 and defined combinations of three distinct intracellular TLRas: TLR3a, TLR9a, and an endosomally restricted TLRa: TLR13a (a single stranded bacterial RNA) (**Figure** **7**a). Analysis of cargo loading revealed equal amounts of antigen across iPEM formulations (Figure S5a, Supporting Information), with control over both the relative and absolute concentration of each iPEM component (Figure 7b,c).

### 2.12 iPEMs Allow Combinatorial Delivery of TLR Agonists and Modular Activation of the Corresponding TLR Pathways

We next measured the ability of iPEMs to co‐deliver antigen and one, two, or three TLR agonists to primary DCs. Using confocal microscopy, we observed a high level of colocalization and juxtaposition of fluorescent signals for Trp2 (green), TLR3a (blue), TLR9a (orange), and TLR13a (red) within cells (Figure 7d). Excitingly, flow cytometry revealed cells treated with iPEMs exhibited uptake profiles that correspond to the composition (i.e., signal levels) of the cargos used to build each iPEM (Figure 7e–k). Notably, these outcomes were observed even with iPEMs containing four immune signals, Trp2‐TLR3a/9a/13a iPEMs (Figure 7d,k). To determine if co‐delivery allows combinatorial control over the relative activation of each TLR pathway, we assessed signaling in three TLR reporter systems. TLR signaling corresponded to the agonist each iPEM contained, and importantly, the relative level of response corresponded to the composition of the iPEMs (Figure 7l–n). Together, these results demonstrate the composition of iPEMs provides control over not just the types of TLRas that are activated, but also the relative levels of signaling in each pathway.

In cancer vaccination the goal is to immunize the host with adjuvants and antigens or neo‐antigens expressed on cancer cells to expand tumor‐specific T cells that also maintain antitumor function in the suppressive tumor environment.^[^ ^39^, ^40^ ^]^ Toward these goals, at least 50 patient trials involving TLRas for cancer indications have been conducted in the past three years.^[^ ^41^, ^42^ ^]^ Human and pre‐clinical studies with these adjuvants have spanned TLRas 2, 3, 4, 7/8, and 9, with polyIC and GpG being intensely studied.^[^ ^43^, ^44^, ^45^ ^]^ Multi‐adjuvant vaccines could have a particularly dramatic impact on cancer because tumor treatments are plagued by antigenic spread and immunosuppression—challenges that polyfunctional responses might help defeat.^[^ ^46^ ^]^ To exploit these pathways, several studies have demonstrated that cancer vaccines formulated with multiple TLRs—particularly combinations of TLR3a, TLR7a, and TLR9a—provide synergistic enhancements.^[^ ^45^, ^46^, ^47^, ^48^, ^49^ ^]^ One key demonstration of the potential of combination adjuvants with cancer vaccination is BCG, a subculture strain of bovine tuberculosis that triggers TLR2, 3, 4, and 9. This approach has shown utility in treating colorectal and bladder cancer, renal cell cancer, and melanoma.^[^ ^50^, ^51^ ^]^ These examples highlight the need for modular adjuvant platforms that allow application of synergistic effects of multifunctional adjuvants, with combinatorial control over the functions of each immune signal.

## 3 Conclusion

Antigen‐specific responses are the defining outcome for vaccines and selective cancer immunotherapies because such outcomes provide efficacy without off‐target effects and the associated toxicity. Emerging research has demonstrated that this goal often requires controlling multiple immune signaling pathway because this integration guides the types of immune responses generated. iPEMs provide a simple platform to program multiple immune signaling pathways for rational design of vaccines or immunotherapies, while mimicking useful features of more complex biomaterial platforms. Ultimately in our studies, activating multiple TLR pathways with iPEM vaccination increased the frequency, trafficking, and proliferation of antigen‐specific T cells in mice. While the scope of this paper was focused on a model antigen, we have recently analyzed a library of defined peptide sequences of varying length, charge, and MHC class to measure binding affinity to nucleic acid based adjuvant; we found that larger peptides with lower charge can still bind nucleic acid adjuvants.^[^ ^52^ ^]^ This shows iPEMs can be used with antigens and adjuvants exhibiting a variety of structural and molecular characteristics. Important future questions for this system include the systemic biodistribution following immunization, and how the pharmacokinetics of TLRas signaling shape the fate of DCs and T cells during disease. For example, it is widely recognized that the immune system plays dual roles in tumor biology with the capacity to support or inhibit tumor occurrence, development, invasion, metastasis, and relapse. Immunotherapy that harnesses one's own immune system to specifically target and kill tumor cells by maximizing the potency through specific sets of immune pathways holds great potential for advancing therapeutic cancer options.

## 4 Experimental Section

### Materials

SIINFEKL peptide (SIINFEKLRRRRRRRRR) was synthesized by Genscript with >98% purity, with a FITC tag on the N‐terminus. Trp2 peptide (SVYDFFVWLRRRRRRRRR) was synthesized by Genscript with a >98% purity, with or without a FITC tag on the N‐terminus. Alexa Fluor 405 NHS Ester (succinimidyl ester) was obtained from Thermo Fisher. PolyIC with a low molecular weight was purchased from Invivogen. CpG oligonucleotide (5′‐TCCATGACGTTCCTGACGTT‐3′) and ORN Sa19 oligonucleotide (5′‐GGACGGAAAGACCCCGUGG‐3′) were synthesized by IDT with a phosphorothioate backbone. LabelIT nucleic acid labeling kits (Cy5 and Cy5) were purchased from Mirus Bio LLC. 4′,6‐Diamidino‐2‐phenylindole (DAPI), wheat germ agglutinin Texas Red conjugate, and paraformaldehyde (4%) were from Life Technologies. CD11c^+^ positive isolation beads were from Miltenyi Biotec. EasySep mouse CD8^+^ isolation kits and spleen dissociation medium were from STEMCELL Technologies. Mouse INF*γ*, IL10, and IL‐6 ELISA kits, ELISA reagents including coating buffer, assay dilution, substrate and stop solution, anti‐mouse monoclonal antibodies for CD80 (APC), CD86 (PE‐Cy7), CD40 (PE), CD11c (APC‐Cy7, PE), B220 (PE‐Cy7), CD3 (APC‐Cy7), and CD8 (APC) were from BD Biosciences.

### Animals and Cells

All animal care and research was carried out using protocols approved and overseen by the University of Maryland IACUC committee in compliance with local, state, and federal guidelines. C57BL/6J mice (female, 6–8 weeks) and OT‐I transgenic mice (female, 6–8 weeks) were obtained from The Jackson Laboratory. Primary CD11c^+^ DCs were isolated from the spleens of naive C57BL/6J mice using a CD11c^+^ magnetic isolation kit, according to the manufacturer's instructions (Miltenyi Biotec). Cells were cultured in RPMI 1640 media (Lonza), supplemented with 10% fetal bovine serum (Corning), 2 × 10^−3^ m l‐glutamine (Gibco), 55 × 10^−6^ m *β*‐mercaptoethanol (Sigma‐Aldrich), 1× non‐essential amino acids (Fisher Scientific), 10 × 10^−3^ m HEPES (Fisher Scientific), and 1× Pen/Strep (Gibco) during primary DCs involved experiments. HEKBlue mouse TLR3 and TLR9 reporter cells were purchased from InvivoGen and cultured following the manufacturer's instruction.

### iPEM Assembly and Characterization

For SIIN iPEMs, calcium carbonate microparticle (MP) templates were prepared as previously described with a slight modification. Briefly, sodium carbonate (Sigma) and calcium chloride dihydrate (Sigma‐Aldrich) were, respectively, dissolved at 0.33 m in water. 10 mL of sodium carbonate was quickly added to equal volumes of calcium chloride dihydrate under stirring (800 rpm) for 30 s. For each batch of iPEMs, MP solution (1:0 1 mL, 3:2 700 µL, 1:1 500 µL, 2:3 500 µL, 0:1 300 µL) was transferred to a 1.5 mL microcentrifuge tube, and aliquots were spun down to collect MPs and washed twice in 1 mL water. To deposit iPEMs, MPs were then resuspended in 1 mL of SIINFEKL solution (0.5 mg mL^−1^ in water) and incubated for 3 min at room temperature. After washing twice in water, the SIINFEKL‐coated templates were incubated in 1 mL of TLRas solution with different compositions but a same total concentration of 0.5 mg mL^−1^ in water for 3 min. The TLRas solution was 100% TLR3a or TLR9a, binary TLRa solution with input compositions of 64:1 (w/w) for 3:2 TLR3a/9a, 16:1 (w/w) for 1:1 TLR3a/9a, and 4:1 (w/w) for 2:3 TLR3a/9a. The resulting particles were washed two more times to complete one bilayer. After coated with three bilayers, the core of particles was removed with two washes in 0.1 m EDTA buffer (pH 6.0). The hollow iPEMs were then washed once in 1× PBS before the iPEMs with different final compositions (w/w), named as 1:0, 3:2, 1:1, 2:3, and 0:1 iPEMs, respectively, were obtained by resuspending in PBS following centrifugation (2000×*g*, 2 min). For Trp2 iPEMs containing combinations of three TLR agonists, 5 mL of sodium carbonate was quickly added to equal volumes of calcium chloride dihydrate under stirring (800 rpm). 5 s later, the stirring speed was swiftly raised to 1200 rpm and the colloidal solution was spun for an additional 40 s to produce the template. For each batch of iPEMs, 300 µL of the MP solution was transferred to a 1.5 mL microcentrifuge tube, and aliquots were spun down to collect MPs before washing twice in 600 µL water. To deposit iPEMs, MPs were then resuspended in 300 µL of Trp2 solution (0.5 mg mL^−1^ in water) and incubated for 5 min at room temperature. After washing twice in water, the Trp2‐coated templates were incubated in 300 µL of TLRas solution with different compositions but a same total concentration of 0.5 mg mL^−1^ in water for 5 min. The TLRas solution was 100% TLR3a, TLR9a, or TLR13a, binary TLRa solution was composed of 8:1 (w/w) for TLR3a/9a and TLR3a/13a, 1:1 (w/w) for TLR9a/13a, or the three classes of TLRa solution were composed of 8:1:1 (TLR3a/9a/13a, w/w/w). The resulting particles were washed twice more to complete one bilayer. After coating with three bilayers, the core of particles was removed with 0.1 m EDTA buffer (pH 6.0). The hollow iPEMs with different composition, named as Trp2‐TLR3a, Trp2‐TLR9a, Trp2‐TLR13a, Trp2‐TLR3a/9a, Trp2‐TLR3a/13a, Trp2‐TLR9a/13a, and Trp2‐TLR3a/9a/13a, respectively, were obtained by resuspending in water following centrifugation (2000×*g*, 3 min).

iPEMs were imaged using fluorescence microscopy (Olympus IX‐83). Diameters were measured by image analysis using ImageJ where iPEMs were thresholded and the particle analyzer function was used to determine the diameter of each iPEM. For imaging TLRas and loading studies, TLRas were fluorescently tagged using a LabelIT nucleic acid labeling kit (Mirus) according to manufacturer's protocol. For loading studies, iPEMs were dissolved in Trypsin/EDTA. Protein loading was assessed by MicroBCA protein quantification assay (Thermo Fisher). TLRa loading was assessed by fluorescence intensity relative to a standard curve.

### Dendritic Cell Uptake Assay

The intracellular internalization of iPEMs in primary DCs was characterized by flow cytometry (FACS CantoII, BD Bioscience) and fluorescence microscopy (Olympus IX‐83). For flow cytometer, primary DCs were isolated as above and were plated in flat bottom 96‐well plate (1 × 10^5^ cells/well) in 0.18 mL DCs culture medium and then treated with fluorescence labeled iPEMs including or PBS as controls. After 18 h incubation, cells were then washed twice in 1% bovine serum albumin (Sigma‐Aldrich) in 1× PBS (FACS buffer) and analyzed by flow cytometry. For fluorescence microscopy to track the localization of iPEMs in cells, DCs were seeded (1 × 10^6^ cells/well) in round‐bottom dishes (35 mm) and were treated with 10 µg CpG overnight to make DCs attached to dish bottom and then received treatments of PBS and iPEMs 3 h. The cells were then gently washed two times with PBS to remove the free iPEM. Cells were then fixed with 4% paraformaldehyde for 15 min at 37 °C and washed twice with PBS. The cells were then stained with Hoechst stain for 5 min. And then cells were washed twice and imaged by fluorescence microscopy under a 63× oil immersion objective. Individual image channels were collected for DAPI (Nucleus), Cy3 (TLR3a), Cy5(TLR9a), and FITC (SIINFEKL) and then merged and analyzed using ImageJ. Cell outlines were drawn using bright field microscopy images of the same cells and overlaying this outline. Images were taken on the same plane as the nucleus to best represent internalization.

### Toll‐Like Receptor Delivery Assays

The intracellular trafficking of iPEMs in primary DCs was characterized by fluorescence microscopy (Olympus IX‐83). For lysosome colocalization studies, DCs were seeded (1 × 10^6^ cells/well) in round‐bottom dishes (35 mm) and were treated with 10 µg CpG overnight at 37 °C to make DCs attach to dish bottom and then received treatments of iPEMs and 2 × 10^−6^ m LysoTracker Red DND‐99 (Thermo Fisher) for 30 min to 3 h at 37 °C. The cells were then gently washed two times with PBS to remove the free iPEMs. Cells were then fixed with 4% paraformaldehyde for 15 min at 37 °C and washed twice with PBS and resuspended in Hoechst stain. To quantify this outcome, image analysis was used to analyze each pixel in the image for the presence of FITC signal from the iPEMs (green) and/or the lysotracker stain signal (red) using the JaCoP plugin in ImageJ.

For TLR staining studies, DCs were seeded (1 × 10^6^ cells/well) in imaging chambers (u‐slide glass bottom #1.5 coverslip, iBidi) previously coated with 0.01% poly‐l‐lysine for 5 min at 37 °C. Cells were treated with 10 µg CpG overnight at 37 °C to make DCs adhere to chamber bottom and then received treatments of iPEMs for 30 min to 3 h at 37 °C. The cells were then fixed and permeabilized with 2% paraformaldehyde for 5 min, followed by several washes in PBS. Paraformaldehyde‐fixed cells were quenched for 10 min with 5 × 10^−3^ m glycine, and permeabilized and blocked with 0.2% saponin in blocking buffer for 30 min at room temperature. Cells were then blocked for 30 min at room temperature in 1% BSA in PBS plus Fc block (blocking buffer). Blocked cells were then incubated with primary antibody (rabbit anti‐mouse TLR3 polyclonal, Thermo Fisher Scientific; or rabbit anti‐mouse TLR9, abcam) in blocking buffer for 1 h at room temperature. Cells were then washed in blocking buffer and incubated for 45 min with secondary antibody (goat anti‐rabbit IgG Alexa Fluor 647) at room temperature before being washed several times in PBS and stored in PBS protected from light before being imaged. Line trace analysis was performed in ImageJ.

For antigen presentation studies, DCs were seeded, fixed, and blocked as described above for TLR staining studies. Wells were then stained at room temperature using a four‐step protocol: 1) 1 h incubation with mouse anti‐mouse H2Kb‐SIINFEKL (Thermo Fisher Scientific) in blocking buffer followed by PBS wash, 2) 45 min incubation with secondary antibody (goat anti‐mouse IgG Alexa Fluor 647) in blocking buffer followed by PBS wash, 3) 1 h incubation with mouse anti‐mouse MHC Class I (H2Kb) (Thermo Fisher Scientific) in blocking buffer followed by PBS wash, and 4) 1 h incubation with streptavidin CF‐594 dye (Biotium) in PBS followed by PBS wash. Samples were then stored in PBS protected from light until imaged. To quantify antigen presentation over time, the corrected total cell fluorescence (CTCF) was calculated using integrated density − (the area of the selected cell of interest × mean fluorescence of the background).

Imaging for TLR staining and antigen presentation studies was performed by confocal microscopy (Zeiss LSM 980 confocal system fitted with an Airyscan 2 module mounted on an inverted Zeiss Axio Observed Z1 microscope with an oil immersion Plan‐Apochromat 63×/1.40 differential interferences contrast (DIC) objective lens (Carl Zeiss, Inc.)). All images were acquired in Airyscan super‐resolution (SR) mode using a 32‐channel GaAsP‐PMT Airyscan detector. Zeiss ZEN 2.3^[^35^]^ software was used for collection and postprocessing of the images. Airyscan processing was done using a 6.3‐strength 3D method. Post‐processing of images was performed in ImageJ 1.53c.^[^ ^53^ ^]^

### RT‐qPCR for Intracellular Cytokine Gene Quantification

Gene expression analysis was performed as previously described.^[^ ^35^ ^]^ Briefly, RNA was isolated from splenic DCs treated with iPEMs, PBS as a negative control, or soluble dose‐matched TLRa as a positive control, using the Quick‐RNA Microprep kit (Zymo Research). Cells were lysed in their wells using a lysis buffer, genomic material was harvested in a silica‐based matrix, and DNA was degraded with DNase I. A Nanodrop 2000c (Thermo Fisher) was used to assess concentration and purity of RNA before the RNA was diluted to 20 ng µL^−1^ in RT‐qPCR grade water (Thermo Fisher). cDNA was reverse transcripted using High‐Capacity cDNA Reverse Transcriptase Kit (Thermo Fisher). qPCR reaction mix was then made using TaqMan Gene Expression Assay probes in TaqMan Gene Expression Master Mix (Thermo Fisher). TaqMan probes used were: glyceraldehyde 3‐phosphate dehydrogenase (Gapdh), Mm99999915_g1; actin beta (Actb), Mm00607939_s1; 18s rRNA (18s), Mm03928990_g1; interleukin 6 (Il6), Mm00446190_m1; interleukin 10 (Il10), Mm01288386_m1; interleukin 12b (Il12b), Mm99999067_m1; interferon gamma (Ifng), Mm01168134_m1; tumor necrosis factor (Tnf), Mm00443258_m1; interferon regulatory factor 3 (Irf 3), Mm00516784_m1; Irf 7, Mm00516788_m1; and nuclear factor kappa B subunit 1 (Nfkb1), Mm00476361_m1. qPCR was performed in a MicroAmp Optical 384‐well reaction plate (Applied Biosystems) with optical adhesive film on a QuantStudio 7 Real‐Time PCR System (Applied Biosystem). Samples were normalized using an average of three housekeeping genes (Gapdh, Actb, 18s). For analysis, data was centered on the PBS control. Analysis and hierarchal clustering were done in Matlab with the clustergram function where the data was standardized for each gene to compare across all groups, and clustering was performed using a single linkage.

### DC Activation

CD11c^+^ splenic DCs were plated as above in 96‐well plate and were treated with iPEMs or PBS as a negative control. After incubation for 18 h, the cells were collected, washed in FACS buffer, and blocked with anti‐CD16/CD32 (BD Biosciences). Cells were then stained with anti‐CD40, anti‐CD80, and anti‐CD86 (BD Bioscience) for 20 min at room temperature, washed two more times as above, and resuspended in DAPI (5 µg mL^−1^) for analysis by flow cytometry.

### T Cell Co‐Culture and Proliferation

CD11c^+^ splenocytes were treated with the above iPEMs, dose‐matched SIINFEKL alone, or dose‐matched SIINFEKL plus dose‐matched TLRas as controls. PBS‐treated cells were used as a negative control. After 24 h, DCs were co‐cultured with CFSE labeled CD8^+^ T cells (3 × 10^5^ T cells per well) that isolated from OT‐I transgenic mice for additional 48 h. Cells were then centrifuged (800×*g* for 5 min), the supernatants were collected for ELISA, and the cells were washed in PBS with 1% FBS. Cells were then blocked with anti‐CD16/CD32 antibody and stained with anti‐CD3 (PE‐Cy7) and anti‐CD8 (APC) for 20 min at room temperature. Finally, cells were washed twice and resuspended in DAPI. T cell proliferation was determined by the mean fluorescence intensity of the CFSE signal among DAPI^−^, CD3^+^, and CD8^+^ cells compared with positive and negative controls.

### Enzyme‐Linked Immunosorbent Assay (ELISA)

Cytokine levels in the supernatants collected from DCs/T cell co‐cultures were quantified by ELISA using mouse IFN*γ* and IL‐6, IL‐10 ELISA reagents (BD Bioscience) according to the manufacturer's instructions. Cytokine concentrations were quantified by comparison to standard curves prepared from known standards.

### Multiple TLR Activation Assays

The TLR signal activity was assessed using HEK‐Blue mTLR3 and mTLR9 cells (Invivogen). Cells were seeded at a concentration of 5 × 10^4^ cells per well followed by treatment with PBS of iPEMs. Cells were incubated in a plate reader for up to 16 h where the absorbance was read at 625 nm every 30 min.

### Lymph Node Analysis by Flow Cytometry

C57BL/6J mice (female, 6–8 weeks) were vaccinated with sham (PBS) or iPEMs (1:0, 1:1, 0:1) subcutaneously at the tail‐base. All iPEM treatments contained 25 µg SIINFEKL and 20 µg total TLRa. Treatments were split into two 50 µL s.c. injections at either side of the tail base. 1, 3, and 5 d after iPEM vaccination, the draining (inguinal) lymph node was harvested and stained for either CD11c, CD40, and CD80, B220 and CD3, or F4/80 and analyzed by flow cytometry.

### Lymph Node Analysis by Immunohistochemistry

C57BL/6J mice were vaccinated with iPEMs as above. Two days post vaccination, the draining inguinal LN was harvested and blocked in OCT compound and analyzed by immunohistochemistry as previously described.^[^ ^31^ ^]^ Briefly, tissues in OCT were quickly frozen using dry ice and kept at −80 °C for long‐term storage. Cryosections were cut at 6 µm in triplicate using a Microm HM 550 cryostat (Thermo Fisher). Sections were fixed with cold acetone/methanol (1:1). Sections were washed with PBS and blocked with donkey serum. Primary antibodies were diluted 1:100–1:200 in PBS and sections were incubated for 1 h in a humidified chamber (Laminin *α*4, Rat clone 775830, RnD; Laminin *α*5, Rabbit polyclonal, Novus Biological; ER‐TR7 (AF 488), Rat Clone MECA‐79, BD); CD3 (Rabbit polyclonal, Abcam; ab49943), B220 (APC), Rat clone RA3‐6B2, eBioScience 17‐0452‐82). Secondary antibodies were applied at 1:400 dilutions for 60 min (Rabbit IgG, Goat polyclonal, Jackson Immunoresearch; Rat IgG Goat Polyclonal, Jackson Immunoresearch; Rabbit IgG, Donkey Polyclonal, Jackson Immunoresearch; Rat IgM, Goal Polyclonal Jackson Immunoresearch). Sections were then fixed with 4% paraformaldehyde, washed in PBS, treated with 1% glycerol and washed in PBS. Sections were mounted with ProLong Gold Antifade (Thermo Fisher). Images were acquired with a Nikon Eclipse 700 (Nikon, Melville, NY) and analyzed with Volocity image analysis software (Perkin Elmer, Waltham, MA). The lymph node cortical ridge area, HEV area and laminin *α*4 and *α*5 area were defined previously.^[^ ^54^ ^]^ The positive staining area percentage was quantified based on at least three independent experiments with 3 mice/group, 2 LN/mouse, 3 sections/LN, 3–5 fields/section.

### Adoptive Transfer Studies

C57BL/6J mice were vaccinated as above with a prime and boost iPEM vaccination on Days 0 and 14, respectively. On Day 15, antigen‐specific CD8^+^ T cells were isolated from the spleens of OT‐I transgenic mice and labeled with CFSE as described above before being resuspended in 1× PBS. 5 × 10^6^ OT‐I CD8^+^ T cells were then adoptively transferred into vaccinated mice via intravenous injection in the tail‐vein. Two days post transfer, the draining and nondraining LNs were harvested and analyzed by flow cytometry as described above.

### MHC‐I Tetramer Staining

C57BL/6J mice were vaccinated as above with a prime and boost iPEM vaccination on Days 0 and 14, respectively. One week post boost, mice were bled submandibular for analysis of antigen‐specific T cells. 100 µL of blood was collected in tubes containing EDTA to prevent coagulation. The blood was then washed in ACK lysis buffer twice before being resuspended in FACs buffer. Cells were washed twice with FACs and blocked with anti‐CD16/CD32 (BD Biosciences). Cells were then stained with SIINFEKL tetramer for 30 min. After 30 min, cells were stained with anti‐CD8 antibody for an additional 20 min at room temperature, washed two more times as above, and resuspended in DAPI (5 µg mL^−1^) for analysis by flow cytometry.

### Antitumor Vaccinations

Mice were vaccinated as above with a prime and boost iPEM vaccination on Days 0 and 14, respectively. Cohorts include an untreated group, iPEMs with the 1:0, 1:1, and 0:1 agonist ratios, and a soluble admixed 1:1 control group where the SIINFEKL antigen did not contain the cationic anchor, thus eliminating complexation of components. iPEM SIINFEKL loading was measured using a microBCA assay and dose‐matched across all treatment groups at each time point. Tumor cells expression the SIINFEKL antigen were inoculated 5 d after boost.

### Tumor Cell Culture, Animal Inoculation, and Volume Calculation

B16‐OVA tumor cells expressing the SIINFEKL antigen were a generous gift from Dr. Kenneth Rock (Dana Farber Cancer Institute). The stocks were tested for contamination using the IDEXX IMPACT 3 service and verified that the cell line was mycoplasma and pathogen‐free. For inoculation, tumor cells were cultured in DMEM supplemented with 10% FBS. The hair on the right dorsal flank of mice was shaved 1 d prior to inoculation with tumor cells to aid injection and monitoring. The mouse flank was cleaned with 70% ethanol then inoculated with 10^5^ B16‐OVA cells in 50 µL of sterile PBS in the dorsal hind flank subcutaneous fat. A 28‐gauge needle was used to inject cells at a slow rate of ≈10 µL s^−1^ to minimize tumor cell shearing. Body weight and tumor volume were monitored once a day following inoculation. Tumors were measured using a digital caliper, measuring along the anterior‐posterior axis and the medial‐lateral axis. The equation *V* = (*W* ^2^ × *L*)/2 was used for volume calculations (https://doi.org/10.1038/laban.254). For survival analysis, a threshold of 75 mm^3^ was used to quantify tumor growth rates. This volume was selected to analyze growth prior to development of tumor ulceration or necrotic tumor core.

### Statistics

Statistical calculations were performed using GraphPad Prism Version 9.4.0 (673). All tests and sample sizes are indicated in figure legends. For the survival analysis, the Log‐rank test was used to generate an exact *p*‐value, which was adjusted using a Sidàk correction as indicated on the figures to account for multiple comparisons. All data points reported in the tumor studies were taken from distinct mice. No data points were removed from studies based on outlier analysis. This is reflected in the traces of individual mice as well as the *n* = 8 for each cohort.

## Conflict of Interest

C.M.J. and R.S.O. are employees of the VA Maryland Health Care System. The views reported in this paper do not reflect the views of the Department of Veterans Affairs or the United States Government. C.M.J. has an equity position with Vaccitech plc.

## Supporting information

Supporting information

## Data Availability

The data that support the findings of this study are available from the corresponding author upon reasonable request.
