# Supplementary material for: Self‐Assembly of Immune Signals to Program Innate Immunity through Rational Adjuvant Design
Source: Adv Sci (Weinh). 2022 Nov 14;10(1):2202393. doi: 10.1002/advs.202202393 (PMC9811447; doi:10.1002/advs.202202393)
Supplement: Supplementary file 1 — Supporting information [file ADVS-10-2202393-s001.pdf]

ADVANCED SCIENCE
Open Access

Supporting Information

for *Adv. Sci.*, DOI 10.1002/advs.202202393

Self-Assembly of Immune Signals to Program Innate Immunity through Rational Adjuvant Design

*Michelle L. Bookstaver, Qin Zeng, Robert S. Oakes, Senta M. Kapnick, Vikas Saxena, Camilla Edwards, Nishedhya Venkataraman, Sheneil K. Black, Xiangbin Zeng, Eugene Froimchuk, Thomas Gebhardt, Jonathan S. Bromberg and Christopher M. Jewell**

Self-assembly of immune signals to program innate immunity through rational adjuvant design

‡Michelle L. Bookstaver[1], ‡Qin Zeng[1], Robert S. Oakes[1,2], Senta M. Kapnick[1], Vikas Saxena[3,4], Camilla Edwards[1], Nishedhya Venkataraman[1], Sheneil K. Black[1], Xiangbin Zeng[1], Eugene Friomchuk[1], Thomas Gebhardt[5], Jonathan S. Bromberg[3,4, 6], and *Christopher M. Jewell[1,2,6-8]

1. Fischell Department of Bioengineering, University of Maryland, 8278 Paint Branch Drive, College Park, MD 20742, USA

2. United States Department of Veterans Affairs, VA Maryland Health Care System, 10 North Greene Street, Baltimore, MD 21201, USA

3. Department of Surgery, University of Maryland School of Medicine, Baltimore, MD, USA

4. Center for Vascular and Inflammatory Diseases, University of Maryland School of Medicine, Baltimore, MD, USA

5. Department of Microbiology & Immunology, The University of Melbourne at the Peter Doherty Institute for Infection & Immunity, Melbourne, Victoria, Australia

6. Department of Microbiology and Immunology, University of Maryland School of Medicine, 685 West Baltimore Street, Baltimore, MD 21201, USA

7. Robert E. Fischell Institute for Biomedical Devices, 8278 Paint Branch Drive, College Park, MD 20742, USA

8. Marlene and Stewart Greenebaum Cancer Center, 22 South Greene Street, Baltimore, MD 21201, USA

‡ These authors contributed equally.

* To whom correspondence should be addressed:

Prof. Christopher M. Jewell
University of Maryland
Fischell Dept of Bioengineering
5110 A. James Clark Hall
8278 Paint Branch Drive
College Park, MD 20742
Office: 301-405-9628
E-mail: cmjewell@umd.edu
Web: jewell.umd.edu

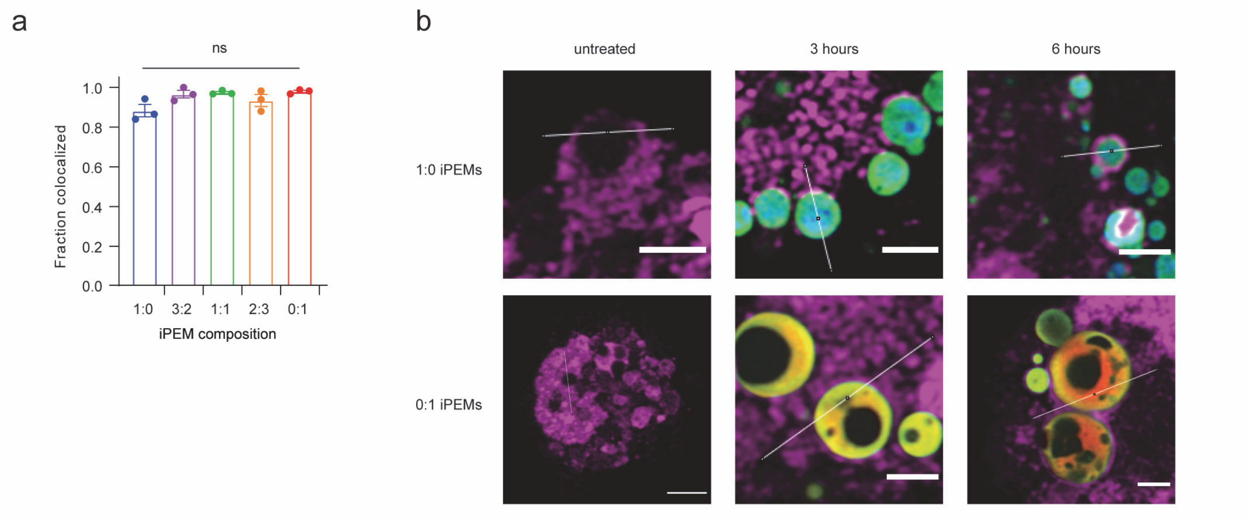

Figure S1. **iPEMs are taken up by primary DCs into endolysosomes**. (**A**) Fraction of iPEM signal co-localized with lysosomes at 3 hrs quantified by image analysis. (**B**) Representative images of line traces used for line trace profile analysis. Scale bar is 2μm. Statistical analysis was done by a two-way ANOVA with a Tukey post test to correct for multiple comparisons. Error bars are S.E.M. n.s. (not significant), * ($p<0.05$), ** ($p<0.01$), *** ($p<0.001$), **** ($p<0.0001$).

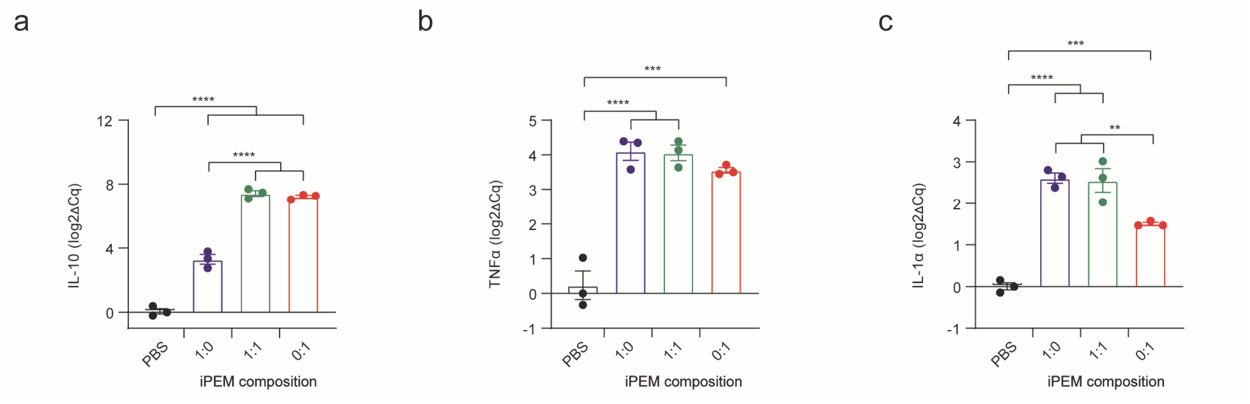

Figure S2. **iPEMs activate TLR signaling pathways as a function of composition**. (**A**) IL-10, (**B**) TNFα, and (**C**) IL-1α expression changes relative to PBS control. n=3 per treatment is an aggregation of n=7 technical treatment replicates. Error bars are S.E.M. Statistical analysis was done by a two-way ANOVA with a Tukey post test to correct for multiple comparisons. n.s. (not significant), * ($p<0.05$), ** ($p<0.01$), *** ($p<0.001$), **** ($p<0.0001$).

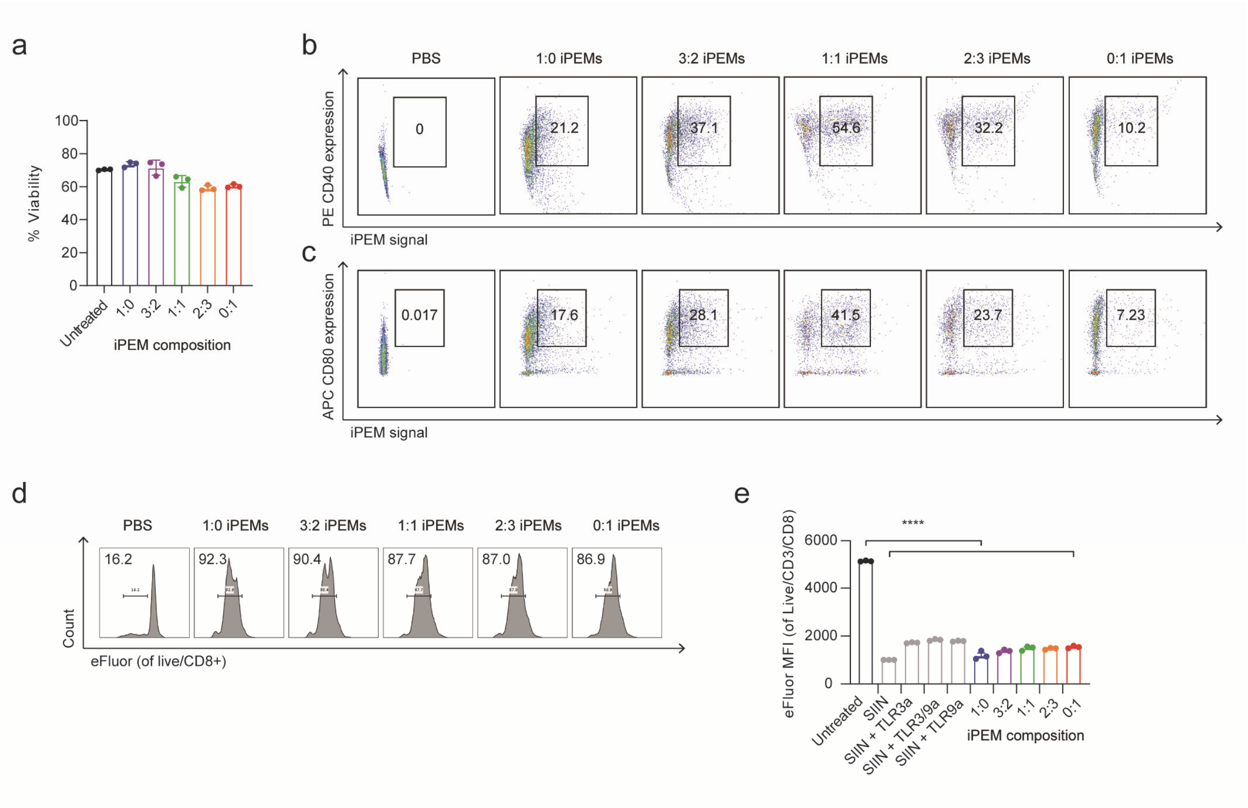

Figure S3. **iPEMs activate DCs as a function of composition.** Representative flow plots of (**A**) viability, (**B**) CD40 expression and (**C**) CD80 expression. (**D**) Representative flow plots of eFluor proliferation assay. (**E**) eFluor MFI showing dilution of eFluor dye following proliferation. n=3 biological replicates for all studies. Error bars are S.E.M. Statistical analysis was done by a two-way ANOVA with a Tukey post test to correct for multiple comparisons. n.s. (not significant), * ($p<0.05$), ** ($p<0.01$), *** ($p<0.001$), **** ($p<0.0001$).

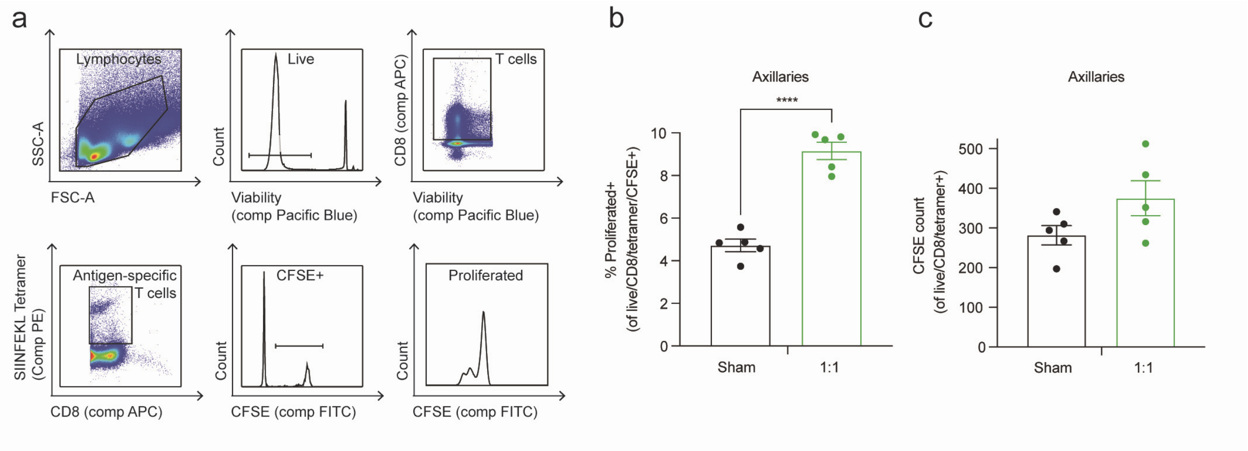

Figure S4. iPEM vaccination alters the trafficking and proliferation of antigen-specific T cells. (**A**) Representative gating scheme for adoptive transfer analysis. (**B**) Proliferation of antigen-specific T cells after prime and boost iPEM vaccinations on Day 1 and Day 15 respectively in the non-dLN (axillary). (**C**) Count of CFSE+ T cells in the non-dLN (axillary) following adoptive transfer. n=5 biological replicates. Statistical analysis was done by a student's two-tailed t test. Error bars are S.E.M. * ($p<0.05$), ** ($p<0.01$), *** ($p<0.001$), **** ($p<0.0001$).

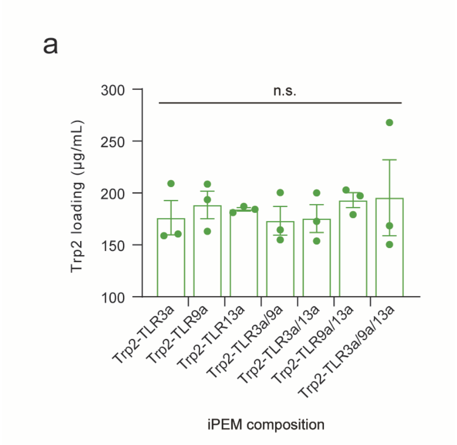

Figure S5. **iPEMs are modularly assembled from multiple TLRas**. (**A**) Loading of Trp2 peptide antigen in iPEM formulations. n=3 biological replicates. Error bars are S.E.M. Statistical analysis was done by a two-way ANOVA with a Tukey post test to correct for multiple comparisons. n.s., not significant.
